# Reappraisal of field dynamics of motor cortex during self-paced finger movements

**DOI:** 10.1002/brb3.186

**Published:** 2013-10-17

**Authors:** Masataka Suzuki, Toshiaki Wasaka, Koji Inui, Ryusuke Kakigi

**Affiliations:** 1 Department of Integrative Physiology, National Institute for Physiological SciencesOkazaki, 444-8585, Japan; 2 Department of Psychology, Kinjo Gakuin UniversityOmori 2-1723 Moriyama, Nagoya, 463-8521, Japan

**Keywords:** Diploe sources, magnetoencephalography, motor cortex, movement-related cerebral fields, somatosensory evoked fields

## Abstract

**Background:**

The exact origin of neuronal responses in the human sensorimotor cortex subserving the generation of voluntary movements remains unclear, despite the presence of characteristic but robust waveforms in the records of electroencephalography or magnetoencephalography (MEG).

**Aims:**

To clarify this fundamental and important problem, we analyzed MEG in more detail using a multidipole model during pulsatile extension of the index finger, and made some important new findings.

**Results:**

Movement-related cerebral fields (MRCFs) were confirmed over the sensorimotor region contralateral to the movement, consisting of a temporal succession of the first premovement component termed motor field, followed by two or three postmovement components termed movement evoked fields. A source analysis was applied to separately model each of these field components. Equivalent current diploes of all components of MRCFs were estimated to be located in the same precentral motor region, and did not differ with respect to their locations and orientations. The somatosensory evoked fields following median nerve stimulation were used to validate these findings through comparisons of the location and orientation of composite sources with those specified in MRCFs. The sources for the earliest components were evoked in Brodmann's area 3b located lateral to the sources of MRCFs, and those for subsequent components in area 5 and the secondary somatosensory area were located posterior to and inferior to the sources of MRCFs, respectively. Another component peaking at a comparable latency with the area 3b source was identified in the precentral motor region where all sources of MRCFs were located.

**Conclusion:**

These results suggest that the MRCF waveform reflects a series of responses originating in the precentral motor area.

## Introduction

Magneto- and electroencephalography (MEG/EEG) are noninvasive neuroimaging techniques that provide a high temporal resolution particularly suitable for investigating the global dynamics of neural activities in the human cortex subserving action, perception, and cognition. In EEG analyses of human voluntary movements, cortical dynamics is illustrated as neuronal information flows among many motor-related regions, particularly in the preparatory period of an intended movement (for a review, see Shibasaki and Hallett 2006). However, the exploration of accurate location of neuronal activities as well as neuronal correlates of control actions using EEG recordings has a physical limitation arising due to the lower conductivity of intervening tissue layers between current sources in the brain and extracranial detectors (Cuffin and Cohen 1979; Rose et al. 1987; Sato et al. 1991). By contrast, MEG offers theoretical advantages that facilitate effective modeling of extracranial electromagnetic fields for source localization. Although MEG fields reflect only the tangentially oriented subset of sources, this problem can be obviated by choosing source responses (Williamson and Kaufman 1987). On this point, MEG has a special advantage in dealing with the components immediately preceding or following the movement onset because both activities are presumed to be tangentially oriented in the anterior or posterior bank of the central sulcus, where neural sensorimotor information is highly integrated.

The MEG recordings of brain activity accompanying a self-paced finger movement show a characteristic pattern of responses, termed movement-related cerebral fields (MRCFs) (Cheyne and Weinberg 1989; Cheyne et al. 1991; Kristeva et al. 1991). Slowly changing neuromagnetic fields termed readiness fields occur preceding movement by 1.0–0.5 sec and are widely distributed over motor-related areas in both hemispheres (e.g., Cheyne et al. 2006). These components are succeeded by a transient response termed motor fields (MFs), which are manifested in the sensorimotor area contralateral to the movement, peaking 40–60 msec before the movement onset (Nagamine et al. 1996). Source modeling studies have provided evidence of the precentral gyrus source location for MF (Cheyne and Weinberg 1989; Cheyne et al. 1991; Kristeva-Feige et al. 1996; Hoshiyama et al. 1997; Cheyne et al. 2006). This component is followed by a rapid succession of two or three components after the movement onset, termed movement evoked fields (MEFs) (Cheyne and Weinberg 1989; Cheyne et al. 1991; Kristeva et al. 1991). The earliest one (MEFI) peaking 30–40 msec after the movement onset has been proposed to reflect reafferent inputs to the cortex from the periphery, arising due to ongoing movements (Cheyne et al. 1997; Kristeva-Feige et al. 1997). Source modeling studies have shown that the source for MEFI is located in Brodmann's area 3b (Kristeva-Feige et al. 1995; Oishi et al. 2004; Cheyne et al. 2006), in area 3a (Cheyne and Weinberg 1989; Onishi et al. 2006), or in both (Kristeva-Feige et al. 1996). Other modeling studies using MEG have reported a precentral gyrus source of the MEFI component (Ganslandt et al. 1999; Woldag et al. 2003; Onishi et al. 2011, 2013). As for the components with a peak latency longer than 100 msec (i.e., MEFII and MEFIII), their cortical generators remain unclear.

In this study, we recorded MRCFs during voluntary finger movements and somatosensory evoked fields (SEFs) following median nerve stimulation, using whole-head MEG recordings with high-density array of sensors. The SEFs have been investigated in great detail to localize early cortical activity of interest for understanding the physiological functions of sensory pathways and to validate the position of the central sulcus as landmark for cortical mapping in neurosurgery. The equivalent current dipoles (ECDs) in the SEFs following electrical stimulation to the skin (Inui et al. 2004) or median nerve (Hari and Kaukoranta 1985; Nakasato et al. 1996; Kakigi et al. 2000) are characteristically localized in contralateral areas 3b, 1, 4, 5, and the bilateral secondary somatosensory (SII) areas, most of which are in the close vicinity of the precentral finger or hand motor area. Thus, precise estimates of the source activities in SEF components can provide us with spatial information to infer the location and direction of dipole sources for each component of MRCFs in the sensorimotor area. Our results based on the multiple source analysis suggested that the MRCF waveform could be modeled by a single source localized in the precentral hand motor region.

## Methods

### Participants

The experiment was performed on 10 (one female and nine males) healthy right-handed volunteers, aged 26–53 years (mean ± SD, 33.8 ± 7.5). All procedures were in accordance with the *Declaration of Helsinki* and approved by the Ethics Committee of the National Institute for Physiological Sciences, Okazaki, Japan. All subjects gave their written consent prior to participation.

### Two experiments

Experiments were conducted in a dimly lit, magnetically shielded room. The subjects were seated with their head firmly fixed using a whole-head neuromagnetometer. Experiments consisted of two parts; recording of MRCFs during finger movements of the right hand, and recording of the somatosensory evoked magnetic fields (SEFs) following median nerve stimulation of the same side. The two experiments were conducted in this order on the same day.

### MRCF experiment

#### Movement

For movement experiments, the forearm was placed comfortably on a table, with the elbow joint flexed 70°. The forearm was pronated to bring the hand into a palm-down position, with all fingers and the thumb flexed naturally. The subjects performed voluntary, impulsive extension with the right index finger at the metacarpophalangeal (MP) joint, followed by immediate return of the finger to the initial resting position. A small plastic plate (1 cm height, 2.0 cm long, 0.3 mm thick) fixed vertically to the tip end of the index finger was placed into a vertical trench (0.6 mm width, 5 cm long in vertical). Cut ends of optical fibers were placed at the same height on both sides of the inner walls of the trench to face each other, such that the light signal was transmittable in open space. When the finger was resting, the plastic plate occluded the switch circuit. Once the finger extended (or moved upward), light was transmitted to switch on the circuit and generate a square pulse, which was used as a trigger signal of averaging in the off-line analyses. The other pair of optical fibers was placed at a height comparable to the fully extended position of the finger, and the corresponding switch circuit generated a trigger pulse when the finger plate occluded the light transmission between these optical fibers. When the index finger was fully extended (0°), therefore, the subject could see the light projected on the plate as a small dot (diameter 5 mm). The subjects were asked to generate an impulsive force to extend their index finger by an amount sufficient to project the light dot on the center of finger plate, and then immediately relax their finger or hand muscles without activation of antagonist muscles. We encouraged the subjects to move the finger in a self-paced manner with an intertrial interval longer than 5 sec. The subjects were asked to keep their gaze on the vertical trench and to minimize the number of blinks and saccadic eye movements across the recording period. To prevent movement overshoot or undershoot, the subjects were allowed a number of practice trials. The recording period was 20 min, in which two rest periods of 1 minute were inserted among three 6-min trial sessions.

#### EMG recordings

A pair of Ag/AgCl electrodes was mounted over the two extrinsic agonist hand muscles (extensor indices muscle, ago1; extensor digitorum muscle, ago2) and one intrinsic antagonist hand muscle (first dorsal interosseous muscle, ant), which act to extend and flex the index finger at the MP joint, respectively. The EMG (electromyography) signals were recorded with a bandpass filter of 0.1–100 Hz online at a sampling rate of 600 Hz.

#### MEG recordings

The MEG signals were recorded as described elsewhere (e.g., Wasaka and Kakigi 2012), with a helmet-shaped 306-channel detector array (Vectorview; Eleka Neuromag Yo, Helsinki, Finland), which comprised 102 identical triple sensor elements. Each sensor element consisted of two orthogonal planar gradiometers and one magnetometer coupled to a multi-SQUID (superconducting quantum interference device) and thus provided three independent measurements of the magnetic fields. The MEG signals were recorded at a 600 Hz sampling rate online with a bandpass filter of 0.1–300 Hz. Raw records for MEG, EMG signals, and trigger pulse signals were all stored continuously on the same computer for off-line analysis.

Prior to the MEG recording, four head position indicator (HPI) coils were placed at specific sites on the scalp. To determine the exact location of the head with respect to the MEG sensors, an electric current was fed to the HPI coil, and the resulting magnetic fields were measured with the MEG sensors. These procedures allowed for alignment of the individual head coordinate system with the MEG coordinate system. The location of the HPI coils with respect to the three anatomical landmarks (nasion and bilateral preauriculas) was also measured using a three-dimensional (3D) digitizer to align the coordinate systems of MEG with magnetic resonance (MR) images, obtained with a 3T MR imaging system (Allegra; Siemens, Erlangen, Germany).

#### Analyses

In the movement task, trials that generated artifacts due to corrective EMG activities before or during movement, or trials that were initiated without an intertrial interval less than 5 sec, were removed following manual inspection on a trial-by-trial basis. Each data set of MEG and EMG signals was time locked to the trigger signal and averaged. The time window of the analysis was from 3000 msec before (−) to 3000 msec after (+) the onset of the trigger signal, and for MEG recordings the prestimulus period from −3000 to −2000 msec was used as the DC baseline. The number of trials used for the analysis averaged 86 (±5) across subjects.

As recorded magnetic fields in each coil are a summation of those from temporally overlapping multiple source activities, a multiple source analysis method has been used to differentiate each source activity (Mauguière et al. 1997; Hari and Forss 1999; Inui et al. 2004; Wang et al. 2004; Jung et al. 2009). We adopt the modeling procedure implemented in BESA 5.1 (MEGIs, Munich, Germany) (Scherg 1990), which allows spatiotemporal modeling of multiple sources over defined intervals. The signal epochs for the source analysis were defined on the basis of global field power (GFP), which was derived by squaring MEG signals for each of two planar-type gradiometers, summing the squared signals together across all channels and normalizing to 100%. The best location and orientation of the dipole source were repeatedly calculated by an iterative least squares fitting algorithm, until the goodness of fit (GOF) expressed as a percentage of the variance of the model to the recorded data reached a maximum.

**Figure 1:**
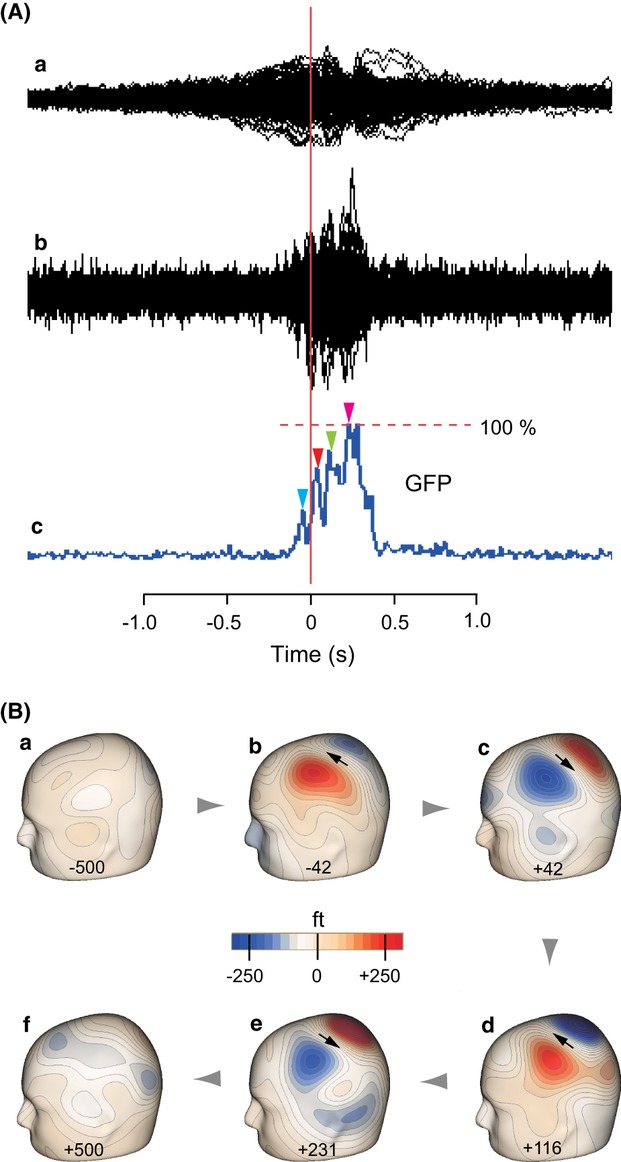
Movement-related cerebral fields following pulsatile extension of the index finger. Data from a representative subject. (A) Superimposed waveforms of all the channels without (a) and with low cut filtering (b). For the latter, the corresponding global field power (GFP) curve is shown in c, in which prominent peaks for specifying motor field (MF) and MEFI–III components are indicated by blue, red, green, and magenta arrowheads, respectively. (B) Six snapshots (a–f) of isocontour maps of evoked magnetic fields in the left hemisphere contralateral to the movement at chosen peaks are indicated by arrowheads in the GFP curve in A-c. The negative values in panels a-b and positive values in panels c-f indicate latency before (−) and after (+) the movement, respectively. Arrows in panels b–e show the location and orientation of estimated equivalent current dipoles (ECDs).

A two-step strategy for localizing generator responses for MRCFs and those in the other regions were applied separately to the averaged waveforms. First, the sensor-level signals were low cut filtered at 2 Hz to separate sharp field components of MRCFs from slow readiness fields, and then the best dipole for explaining the major magnetic field components was modeled at each peak appearing in the GFP curve using a single-dipole analysis (Fig. 1A). To achieve this, the GFP curve was divided into four time windows: a period of 100 msec before the movement onset (−100 ∼ 0 msec), a period of the first 80 msec after movement onset (0 ∼ 80 msec), second 90 ∼ 180 msec, and third 200 ∼ 300 msec, each of which was expected to involve one prominent peak with comparable latencies reported for MF and MEFI–MEFIII, respectively. In each epoch, however, the number of peaks in the GFP curve was often more than one or the peak itself was not apparent due to contamination by noise, both leading to difficulty in discriminating which peak is most appropriate for modeling each component of MRCFs. In such cases, a principal component analysis (PCA) was repeatedly applied to each time bin of 10 msec duration in the GFP curve in the corresponding time window. The time bin of greatest variance was used to model one dipolar magnetic field pattern in the corresponding epoch. Differences in the spatial positions or directions of four dipole sources in MRCFs were assessed using analyses of variance (ANOVA). Next, sources responsible for the activity of the other brain regions were added to the model by using a multidipole analysis (Inui et al. 2004), retaining the solutions for all MRCF components. We continued to add sources to the model until a GOF value >80% was obtained. The locations and orientations of sources were compared in a 3D space.

### SEF experiment

#### Stimulation

To elicit SEFs, transcutaneous electrical stimulations were applied to the right median nerve at the wrist using a conventional bipolar felt tip electrode 0.9 mm in diameter with a distance of 23 mm between the anode and cathode (Kakigi et al. 2000). The stimulus, a current constant square wave pulse with 0.5 msec duration, was delivered at an interval of 3 sec. The current intensity was adjusted to a level comparable to the motor threshold (6.0 ± 1.3 mA). For each subject, the data for 100 stimuli were collected consecutively.

#### Analyses

Procedures for SEF recordings were the same as those for MRCFs. The same standard procedure for source analysis as described in the second step described above was used to estimate source activity in the SEF data (Mauguière et al. 1997; Hari and Forss 1999; Inui et al. 2004; Wang et al. 2004; Jung et al. 2009). The time range of the source analysis was from 100 msec before to 250 msec after the onset of the stimulus. The data for 100 msec before the stimulus were used to calculate the baseline. The major peaks in the GFP curve were specified, retaining the dipole solutions determined earlier. We considered that when the residual variance (100% – GOF%) was less than 10%, the adaptation of the dipoles would be effective. The differences in dipole locations or orientations among all possible combinations of components in MRCFs and those specified in the SEF waveform were assessed one by one using the unpaired t-test.

## Results

### Spatiotemporal pattern of MRCFs

Figure 1A-a shows a typical MEG record during the finger movement of a representative subject that consists of slow readiness fields and then several sharper components. To analyze the neural origin of the latter, a high-pass filter was applied (Ab), such that several peaks could be clearly identified in the corresponding GFP curve (Ac). Snapshots of the isocontour map at these selected peaks (indicated by arrowheads in Ac) showed that that the field topography in the left sensorimotor region contralateral to the side of movement was sequenced with a series of apparent dipolar patterns of activation, changing their orientations from anterior–superior to posterior–inferior alternately (Fig. 1B). The first peak appeared at a latency of 42 msec before the movement onset in the superior–anterior direction (b) and the second one was at 42 msec following the movement onset in the inferior–posterior direction (c). Thereafter, two peaks followed with alternating dipolar pattern of activities at 116 (d) and 231 msec (e). Taking the latencies of these peaks into account, it is apparent that the first-to-fourth peaks in the GFP curve (Ac) reflect four components of MRCFs, that is, MF, MEFI–MEFIII, respectively. In the following, the sources responsible for MF, MEFI, MEFII, and MEFIII field components are named smf, sm1, sm2, and sm3, respectively.

**Figure 2:**
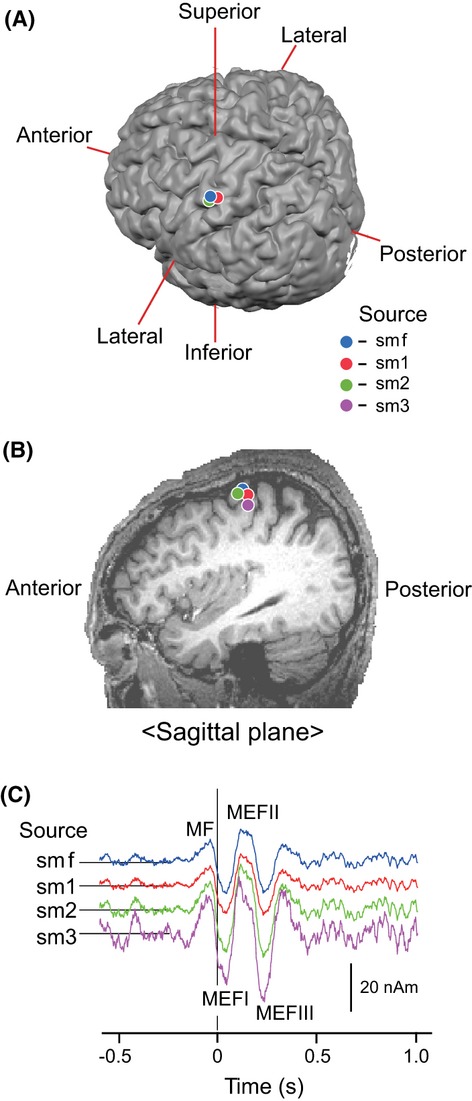
Spatiotemporal characteristics of source response modeled for movement-related cerebral fields (MRCFs). (A) Superpositions of four dipole sources (smf, sm1–sm3) on an MR image in posterior/superior oblique view. (B) The same superpositions of four dipoles sources in the sagittal plane view through the motor cortex region in the left hemisphere. Note that three or four plots seem to locate in nearly the same position in the posterior crown (smf, sm1, and sm2) in A or the wall of the precentral gyrus (smf, sm1–sm3) in B. (C) Comparison of the time course of source strength among four different dipole sources, smf (blue), sm1 (red), sm2 (green), and sm3 (magenta).

As predicted in the spatiotemporal pattern of field distribution in Figure 1B, results of a single-dipole analysis at each peak in the GFP curve showed that all four dipoles (smf, sm1–sm3) had almost identical orientations and were located in a similar region in the hemisphere contralateral to the movement. Figures 2A and B show superimpositions of these sources' locations on MR images of the corresponding subjects, showing that all sources were located in the posterior crown or posterior wall of the precentral gyrus in the left hemisphere, respectively. Figure 2C shows the source strength as a function of time for the four corresponding dipoles. All sources share nearly the same time course of waveform across the movement times, with minor discrepancies in peak times. Correlation analyses of the time courses of activities between all possible pairs among four sources showed high coefficient values more than 0.98 (*P* < 0.001, *n* = 1200 for all) in all subjects, supporting the view that all the MF, MEFI, MEFII, and MEFIII components can be explained well by the same dipole.

**Figure 3:**
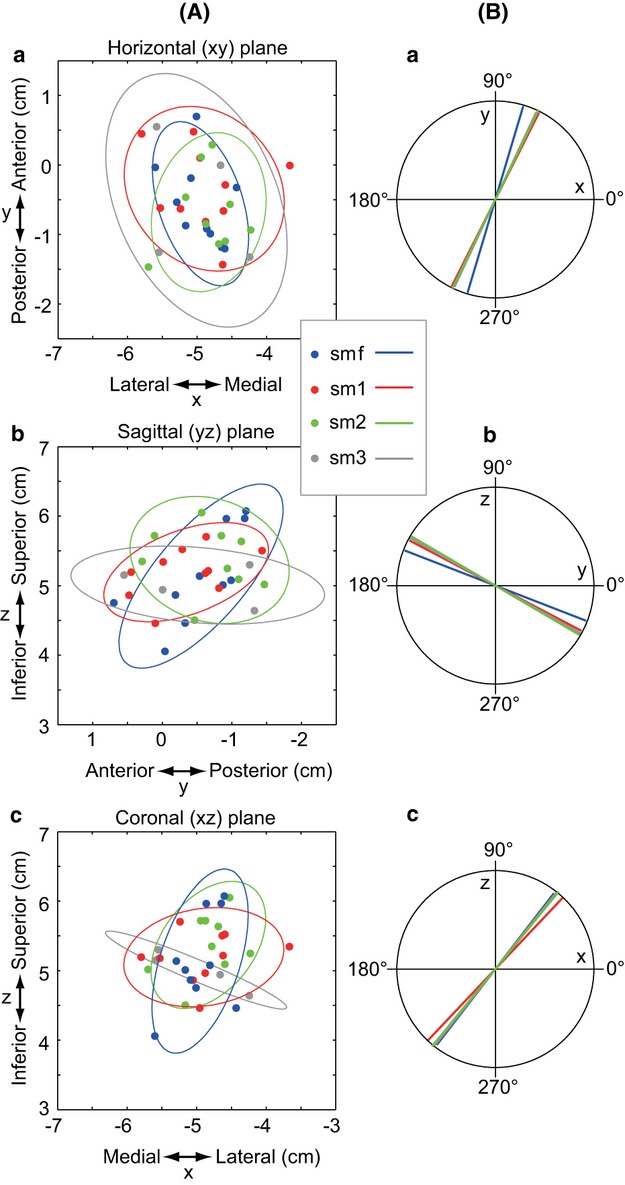
Spatial locations and orientations of four sources in the movement-related cerebral fields (MRCFs). (A) Plots for the locations of four independent sources (smf, sm1–sm3) in MRCFs in all subjects, in horizontal (a), sagittal (b), and coronal (c) planes of magnetoencephalography (MEG) coordinates. In each source, an ellipse represents a 95% confidence limit (*z*-value: 1.96) for plots in each of three orthogonal planes. (B) Orientation of four sources, averaged separately, in three orthogonal planes, corresponding to the sources in the left panels.

Similar procedures were applied to data for the remaining subjects. Figure 3A shows plots of the locations for smf, sm1–sm3 of all subjects, depicted in three orthogonal planes of MEG coordinates. The smf and sm1 were confirmed across subjects, whereas those for sm2 and sm3 were identified in nine and four subjects, respectively. No difference was found in source locations in the medial–lateral (*x*) direction (*F* = 0.45, *P* = 0.72), anterior–posterior (*y*) direction (*F* = 0.16, *P* = 0.93), and superior–inferior (*z*) direction (*F* = 0.59, *P* = 0.63). Similarly, the source orientation did not differ significantly among the four dipoles. Figure 3B illustrates this in three orthogonal planes. The orientations of the four components averaged 67 ± 11°, 154 ± 9°, and 50 ± 10° in the horizontal (*xy*), sagittal (*yz*), and coronal (*xz*) planes, respectively. In each plane, no difference was found in orientation among the four components (*F* = 1.91, *P* = 0.15 in a; *F* = 1.96, *P* = 0.14 in b; *F* = 0.64, *P* = 0.66 in c). These consistencies of source profiles in terms of locations and orientations suggest that a series of prominent peaks of MRCFs could not be ascribed to the manifestation of separate source activities.

### Relation to EMG activities

**Figure 4:**
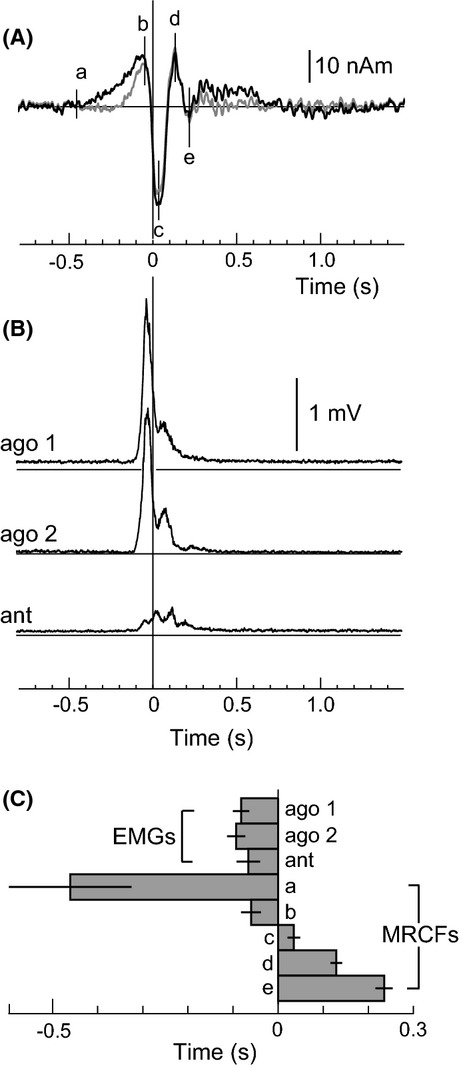
Temporal relationship between movement-related cerebral fields (MRCFs) and EMGs. (A) MRCF waveform modeled based on the parameters of smf, averaged across subjects. Black and gray lines represent unfiltered and filtered responses, respectively. The vertical line a represents the onset time of readiness fields, and peak latency for motor field (MF), MEFI, MEFII, and MEFIII are indicated in alphabetical order from b to e, respectively. The onset latency of the readiness field and peak latencies of MEFs were derived from the records of unfiltered and filtered MEG responses, respectively. (B) Rectified EMG signals recorded from two agonist muscles (ago1 and ago2) and one antagonist muscle (ant), both of which are time locked to the trigger pulse, and averaged across subjects. (C) Temporal relationship between the onset latency of activation of three muscles and readiness fields (a), and peak latency for MF (b), MEFI (c), MEFII (d), and MEFIII (e).

The temporal relationship between MRCFs and EMGs is shown in Figure 4. The MRCF waveform modeled from smf (A) and rectified EMG signals (B), both time locked to the trigger pulse, was averaged across subjects. In Figure 4A, when the smf dipole was applied to model original, unfiltered MEG signals, the mean onset latency of the MRCFs across subjects was −461 (±136) msec (see solid line), but we did not approach this measure anymore; instead, the high-pass filtered MEG signals were used to effectively extract and model the source activities of MF and subsequent MEFs components (see gray line). As shown in Figure 4C, the peak times of MF and MEFI-III components averaged −60 ± 21, 38 ± 14, 129 ± 13, and 235 ± 19 msec, respectively. They were statistically different (*F* = 2.92, *P* < 0.001), such that the temporal order of four peaks was robust, regardless of the lack of an MEFII or III component in some subjects.

As shown in Figure 4B, two agonist muscles initiated EMG activities just before the peak times of MF components in Figure 4A. The EMG onset times of ago1, ago2, and ant averaged −82, −93, and −66 msec, respectively, and were not different (*F* = 3.55, *P* = 0.08) (Fig. 4C). Clear EMG activity of the antagonist muscle (ant) was very limited or, if present, was coactivated with agonist EMGs without phasic bursts following agonist bursts (Fig. 4B). This implies that the finger extension was decelerated without apparent excitation of the antagonist muscle following agonist bursts, and thus the recovery of the finger position toward its initial state was ascribed to a combination of opposing forces due to gravity, elasticity of the skin, and passive elements of the tendon or muscles. On this point, it can also be said that the subjects executed the “pulsatile” task precisely without apparent contribution of their antagonist muscles.

### SEF sources

**Figure 5:**
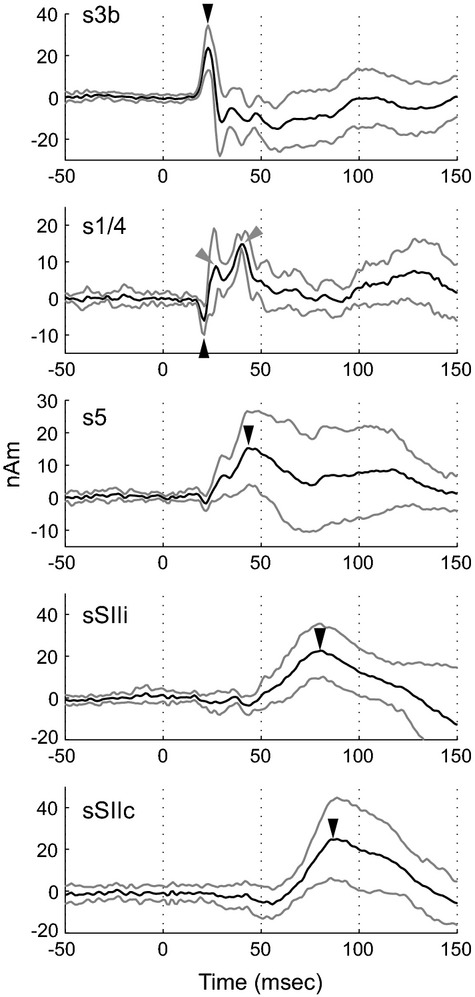
Grand-averaged source strength waveforms for cortical sources in somatosensory evoked fields (SEFs). From top to bottom panels, solid line represents averaged waveforms for s3b, s1/4, s5, sSIIi, and sSIIc across subjects are shown, whereas the thin lines represents ±SD. In each panel, a closed arrowhead indicates the peak time of initial source activities. Note in panel of s1/4, two additional peaks are indicated by gray arrowheads.

The cortical sources for SEFs following median nerve stimulation were investigated to determine the spatial position and orientation of sources modeled for MRCFs. Figure 5 shows the time course of each source activity, averaged across subjects. The earliest, phasic component was specified at a peak latency averaging 21 msec (see arrowhead). The superimposition of this source on each subject's own MR images showed that the best source location to explain the field pattern of this latency was located in the posterior bank of the central sulcus, corresponding to area 3b, and thus termed s3b. Next, magnetic fields due to the s3b were subtracted from the recorded magnetic fields, and the second-best dipole was searched among the residual fields that had a peak at around 25 msec. The superimposition of this source on MR images showed that it was located in the anterior crown of the postcentral gyrus or posterior crown of the precentral gyrus, and thus termed s1/4. Although the source strength waveform of this source showed a clear peak at 25 msec (indicated by a gray arrowhead), there was an additional earlier component peaking at 20 msec (a black arrowhead) and a later one peaking at 41 msec. In a similar way, s5, the source in area 5, and then sSIIc and sSIIi, the sources in the secondary somatosensory areas of left and right hemispheres, respectively, were specified.

### Spatial relationship of sources for MRCFs and SEFs

**Figure 6:**
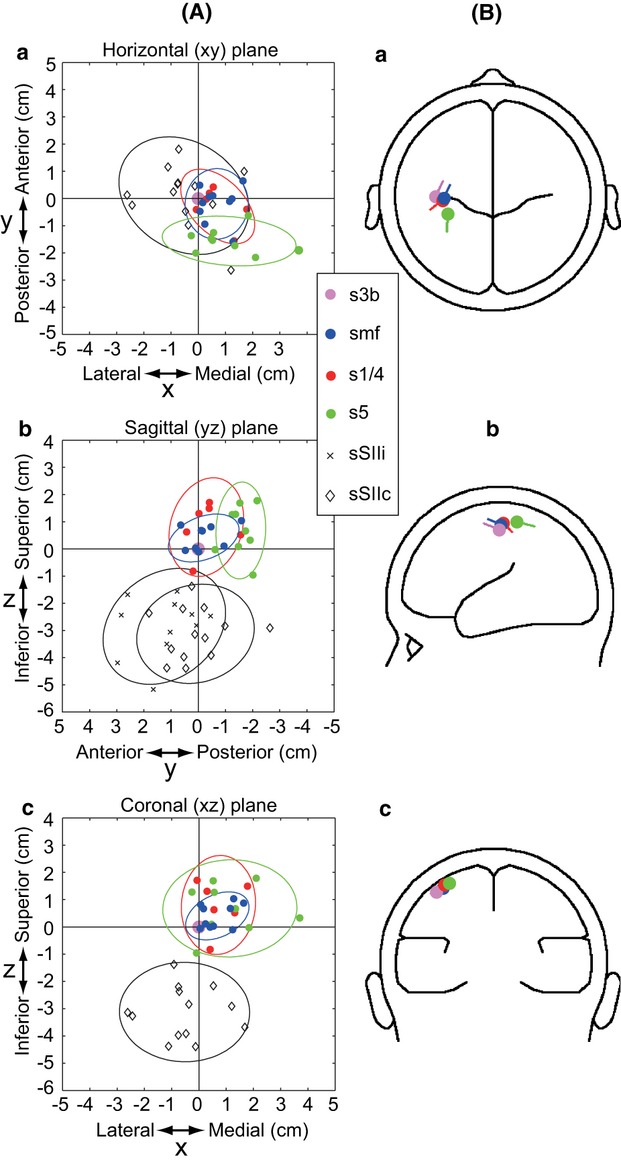
Simultaneous representation for spatial locations and orientations of four independent sources in somatosensory evoked fields (SEFs) and smf in MRCFs. (A) Plots for locations of all sources in SEFs (i.e., s1/4, s5, sSIIi, and sSIIc) and of smf are presented relative to the positions of s3b (defined as the cross-point of vertical and horizontal lines in the figure) in three orthogonal planes; from top to bottom, horizontal (a), sagittal (b), and coronal (c) planes. In each panel, an ellipse represents a 95% confidence limit (*z*-value: 1.96) for corresponding plots of source location in each of three orthogonal planes. (B) Grand-averaged source locations and orientations of four independent sources in SEFs and smf. The same sources to those depicted in A are averaged and depicted. In each source, the orientation of the current is represented by the line segment embedded in the plot for the location of the corresponding source. In three anatomical planes, source orientations are represented as their projection on three orthogonal planes, that is, horizontal (*xy*), sagittal (*xz*), and coronal (*yz*) plane, in panels a to c, respectively.

The anatomical locations of sources for MRCFs and SEFs were transformed to a common coordinate system relative to the location of area 3b sources (s3b). Figure 6 shows this comparison in three orthogonal planes. First, the location was compared among three SEF sources, s3b, s1/4, and s5. As shown in Figure 6B, they were arranged medioposteriorly in this order in the postcentral region, and could be separated statistically in at least one coordinate in the comparisons of s3b and s5, or of s1/4 and s5 (Table 1). Next, the location was compared between SEF and MRCF sources. All the MRCF sources' locations were statistically distinct from those for s3b and s5, whereas all components for MRCFs did not differ significantly from s1/4 in all axes (Table 1). The latter finding suggests that the source locations of s1/4 and four components of MRCF are nearly consistent in 3D coordinates, as can be seen in Figure 6A and B. This might be contrasted with the significant separations between s3b and each of the MRCF sources in the *x*-axis or those between s5 and MRCF sources in the *y*-axis, respectively (Table 1). The same statistical analysis was repeated by defining the spatial position of MF as the origin in 3D MEG coordinates. A similar tendency for the spatial relationships among positions of s3b, s1/4, s5, and all sources of MRCFs was reconfirmed (data not shown).

**Table 1:** Difference in 3D location among components of MRCFs and SEFs

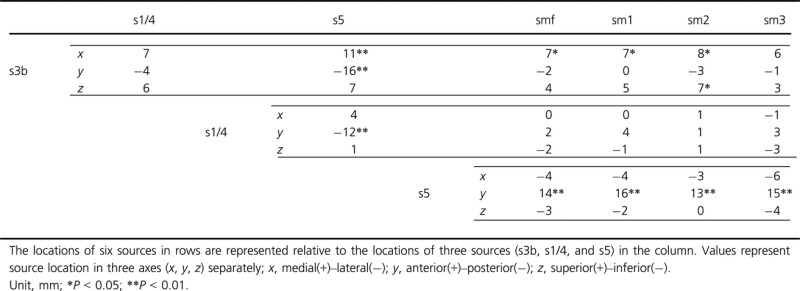

Figure 6B illustrates the comparison of source orientations among smf, s3b, s1/4, and s5 in three orthogonal planes. Results of the same comparison of orientations of all sources in the MRCFs and those in the SEFs are summarized in Table 2. As shown in Figure 6B, s3b shows a quite similar orientation to those of smf in all planes. Thus, it can be said that the s3b and smf sources are similar in orientation but different in location (see Table 1), which is well explained by two groups of neurons in the postcentral and precentral gyri, respectively. In contrast, s1/4 was localized in nearly the same position for all sources of MRCFs (Fig. 6A) (see Table 1) with an apparent discrepancy in orientation in the horizontal and sagittal planes (Table 2). Assuming that both sources are of the same neuronal population in the precentral region, they are expected to have similar directions. Our results suggest, therefore, that the sources for s1/4 and those for MRCFs are based on the activations of different neuronal populations in the same precentral motor region. In addition, differences in source orientation between s5 and components of MRCFs were apparent in the horizontal and sagittal planes (Table 2).

**Table 2:** Difference in orientation among components of MRCFs and SEFs

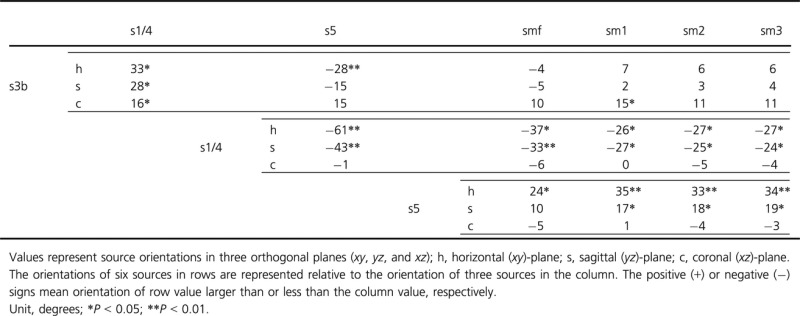

### Sources in other brain regions

The magnetic fields responsible for the MRCFs were subtracted from the recorded magnetic fields. In the residual fields, components showing a dipolar pattern of activity were explored over the hemispheres. Table 3 summarizes these results. The regions related to visual processing (or movement monitoring) or somatosensory processing that might be attributable to the planning and execution of movement exhibited dipolar pattern of activations across movement time. These responses consist of slow premovement dipole activities with nearly the same onset times as those observed for the MF component. Thereafter, however, no phasic alternation of peaks like those observed in MRCF waveforms was observed, excepting for a response that often appeared in the occipital region as seen in panel d in Figure 1B. This observation could be extended to those observed in the dipolar pattern of activation in the ipsilateral sensorimotor area, leading to a limitation for the number of dipoles specified in this area (Table 3).

**Table 3:** The number of dipole source in brain areas of two hemispheres

	Movement		Stimulation	
		
Brain area	Contralateral	Ipsilateral	Contralateral	Ipsilateral
SI/MI	33 (10)	8 (8)	6 (6)	
3b			10 (10)	
PPC	3 (3)	2 (2)	9 (9)	
SII	13 (10)	11 (10)	13 (10)	13 (10)
PM	4 (4)	4 (4)		
TPJ	3 (3)	3 (3)		
STS	6 (6)	3 (3)		
SFG	1 (1)	2 (2)		
SMA	4 (4)^1^			
ACC	6 (6)^1^			
PC	4 (4)	3 (3)		
SC (V1)	7 (7)	5 (5)		
ESC	8 (8)	7 (7)		

Values in parenthesis mean the number of subjects. SI/MI, primary sensorimotor area. In SI/MI in contralateral side, the number of dipole sources is the sum of MF, MEFI, MEFII, and MEFIII components. 3b, area 3b. Note sources specified in area 3b were cited independently from areas involved in SI/MI. SII, secondary somatosensory area. In SII, for both movement and stimulation data, the same subjects showed one to three EDSs in both hemispheres. PPC, posterior parietal cortex; PM, premotor area; TPJ, temporoparietal junction; STS, superior temporal sulcus; SFG, superior frontal gyrus; SMA, supplementary motor cortex; ACC, anterior cingulate cortex; PC, precuneus; SC, striate cortex (visual area 1); ESC, extrastriate cortex.

1 Dipole source is located in the mesial portion of the cortex.

## Discussion

In this study, neural sources of MRCFs generated during a pulsatile extension of the index finger were modeled to ascertain whether multiple sharp components originate from independent source activities. Two to four sources (i.e., smf, sm1–sm3) were modeled independently across subjects. The position, orientation, and time-varying patterns of these sources were compared to those obtained for the components in the SEF data. We found that all dipole sources for MRCFs were located in the same precentral region, oriented in the same direction in the cortical space, and exhibited the same time-varying wave profiles over the movement time. These led us to suggest that there is no specific reason to deal with the four components of the MRCF waveform independently, but rather that all components of MRCF originate from the precentral motor area.

### Readiness field and MF

Cortical activity preceding voluntary movements has been documented in neurophysiological studies in humans (Kornhuber and Deecke 1965; Barrett et al. 1986; Jahanshahi et al. 1995; Richter et al. 1997; Wildgruber et al. 1997) and monkeys (Gemba et al. 1980; Sasaki and Gemba 1981). In humans, high-resolution scalp EEG recordings suggest that brain activation during preparation for self-paced movement in humans initiates 1 ∼ 2 sec before the movement onset in the supplementary motor area (SMA), pre-SMA, premotor cortex, primary motor cortex, and the anterior cingulate cortex (Cui et al. 2000). Involvement of these cortical areas was confirmed by subdural recordings (Ikeda et al. 1992, 1995; Yazawa et al. 2000). In the raw records of MEG signals without high-pass filtering of signals, we also observed premovement field activities in these cortical regions, but the slow shift (readiness fields) beginning earlier than 1.0 sec before the movement onset was not manifested anywhere over the cortex (Fig. 1A; see also Fig. 4A). This might be attributable to the spatial orientations of MEG sensors that are insensitive to a dipole with an intracellular current radial to the brain surface.

Shibasaki and Hallett (2006) subdivided the readiness potential into two components. The first is a slow, negative potential preceding onsets of self-paced movements by 1 ∼ 2 sec (e.g., Barrett et al. 1986) covering many regions in each hemisphere, whereas the second one is observed mainly in the sensorimotor region contralateral to the movement and rises more steeply 0.5 sec before the movement onset (e.g., Ikeda et al. 1992). The second component peaking just before the movement onset reflects MF activity in MEG records (Nagamine et al. 1996). According to this scheme, the MF activity we observed in the high-pass filtered responses (e.g., Fig. 1A-b) may partly involve the early component similar to that recorded in EEG studies, but mainly reflects the spatiotemporal pattern of the latter component over the sensorimotor area in the hemisphere contralateral to the movement.

### Sources composing MRCFs

We found all sources of MRCFs to be in close vicinity of the central sulcus in group data (Fig. 3). Among these, the mean source location for the MF (smf) was found to be 7 mm medial to s3b in the postcentral gyrus (Table 1). It is widely accepted that MF is generated in the primary motor cortex (area 4) in the anterior bank of the central sulcus (Cheyne and Weinberg 1989; Kristeva et al. 1991; Ball et al. 1999; Cheyne et al. 2006). Of more importance in our findings is that sources of MF and of the subsequent three components (MEFI–MEFIII) are all localized at nearly the same portions of the precentral gyrus where finger and hand motor areas locate (Yousry et al. 1997).

The source of MEFI has been proposed to reflect either of two components in the posterior wall of the central sulcus or deep in the central sulcus, reflecting activation due to tactile or proprioceptive afferent inputs to areas 3b or 3a, respectively (Kristeva-Feige et al. 1995, 1996, 1997; Oishi et al. 2004; Cheyne et al. 2006). However, the removal of cutaneous inputs does not decrease the MEFI response, but rather enhances it (Kristeva-Feige et al. 1996), suggesting that proprioceptive inputs to area 3a also contribute to the generation of MEFI, as supported by later studies (Mima et al. 1996; Woldag et al. 2003). In our results, the peak latencies of MEFI averaged 38 msec after the movement onset (Fig. 4), which is comparable to the earliest cortical response following passive movement without muscle contraction. Mima et al. (1996) have shown that when the index finger is passively extended without muscle activation, an initial EEG response elicited in the sensorimotor region peaks at a latency of 35 msec. They have suggested area 3a involvement for this response, as selective nerve blocking of muscle afferents using ischemia abolishes it. Using the same procedure with MEG recordings, however, Onishi et al. (2013) have found that an initial magnetic response elicited in the precentral motor region peaks at 36 msec. They have also shown that both the peak latency and the location of ECD of this response were similar to those of MEFI following active movement. In an intracranial recording study in humans (Papakostopoulos et al. 1974), the peak latency of activation in the motor region in response to passive finger displacement was 34 msec. Thus, it is likely that MEFI peaking at 38 msec after the movement onsets in this study reflects muscle afferent inputs, probably arising due to the stretching of antagonist muscles during finger extensions (Onishi et al. 2013). The contribution of the muscle afferent in shaping MEFI activity may be ascribed to its excitatory effect on the motor cortex neurons, through direct projection from the thalamus (Horne and Tracey 1979; Lemon and van der Burg 1979; Asanuma et al. 1980; Tracey et al. 1980; Butler et al. 1992), or indirectly by way of area 3a to area 4 (Zarzecki et al. 1978; Ghosh and Porter 1988; Avendano et al. 1992). Widener and Cheney (1997) have supported the former suggestion based on the finding that the responses of primary somatosensory neurons in behaving monkeys, including area 3a neurons, to torque pulse perturbations are relatively weak or absent. In our results, taking both the source location and the response latency of the MEFI response into account, it is suggested that it reflects activation in area 4, although area 3a involvement cannot be neglected.

### Implications from SEF studies

Supporting evidence to show that all components of MRCFs are of precentral motor cortex origin can be found in the spatiotemporal pattern of source responses specified in the SFE data. First, the latencies of the SEF sources reflect the time necessary for signals to reach corresponding regions of the cortex. The source for the first response s3b, peaking at 21 msec, was located in the posterior bank of the central sulcus, corresponding to area 3b. This finding was consistent with previous somatosensory evoked potential (SEP) and SEF studies demonstrating that the earliest cortical response to median nerve stimulation originates from area 3b in humans (Wood et al. 1985; Allison et al. 1989; Kawamura et al. 1996; Papadelis et al. 2011; Frot et al. 2013) and monkeys (McCarthy et al. 1991). Second, we showed that the exact origin of s1/4 was in the same precentral motor cortex region to all sources of MRCFs (Table 1). As for the involvement of precentral sources of SEFs, care should be taken because there is still debate about the origin of the response(s) occurring at nearly comparable times or a few millisecond later (<2 ∼ 3 msec) to the 3b response, which has been assigned either to area 4 or to area 1. Allison and coworkers used subdural grid recordings of patients undergoing epilepsy surgery and suggested that the P22 component would most likely originate from area 1 (Wood et al. 1985; Allison et al. 1989; see also Baumgärtner et al. 2010), whereas Jung et al. (2008) localized the P22 dipole source in area 4, using an EEG dipole source analysis. More recently, Frot et al. (2013) approached this problem using intracortical recordings of potentials following median nerve stimulation in humans. They have clearly shown that both the precentral (area 4) and postcental (area 3b) responses occur at the same latency of 22 msec, but with an apparent phase reversal across the central sulcus. This indicates the presence of area 4 responses due to median nerve stimulation.

Using multiple source modeling of magnetic fields following transcutaneous stimulation of the hand, Inui et al. (2004) succeeded in modeling three independent components of field responses in areas 3b, 4, and 1 near the central sulcal region. They showed the peak latency of area 4 activity to be 21 msec, which was nearly simultaneous to that of area 3b (20 msec), while other one peaking at 25 msec represented activity originating from area 1 (see also Papadelis et al. 2011). In our analysis, the latency of the first peak of s1/4 averaged 20 msec, being comparable to the peak latency of area 4 rather than that of area 1 reported by Inui et al. (2004). According to Inui et al. (2004), moreover, the relative locations of area 1 were more medial (9 mm), superior (12.7 mm), and posterior (7.2 mm) than the area 3b source, being around the anterior crown of the postcentral gyrus. Our estimates for the s1/4 location were 7 mm medial, 6 mm superior, and 4 mm posterior relative to 3b location (Fig. 6; Table 1). The major difference across all axes in these two studies was manifest in the superior–inferior (*z*) direction: our estimate for s1/4 position was 6.7 mm inferior relative to the area 1 source location estimated by Inui et al. (2004), which corresponds to the deep fissural part of the precentral sulcus where all components for MRCFs in our data were located (Fig. 6; Table 1). This suggests that the first component of s1/4 in our study reflects the source response originating in area 4, whereas the following peak at latency of 25 msec or more may reflect a contamination of source activity in area 1, which had been successfully separated from the area 4 component by Inui et al. (2004; see also Figs. 5 in Frot et al. 2013). According to Frot et al. (2013), sources for areas 4 and 3b, separated by the central sulcus, are located side by side in the lateral–medial direction (see also Fig. 5 in Kawamura et al. 1996). The same scheme can be found in our data (Fig. 6B-a), and thus strengthen our proposal of a precentral origin of s1/4. The third source s5 in the postcentral gyrus was in its caudal-most part around the intraparietal sulcus at a latency of 50 msec, probably corresponding to area 5 in agreement with previous MEG studies (Forss et al. 1994; Hoshiyama et al. 1997; Inui et al. 2004).

A few previous MEG studies on decomposing MRCFs have proposed that the source of MEFI is of postcentral origin, perhaps in area 3b, and reflects feedback from the periphery (Oishi et al. 2004; Cheyne et al. 2006), leading to our speculation of the commonality of source locations for MEFI during movement experiments and area 3b in SEF experiments. However, the source locations we specified differed substantially, mainly in the medial–lateral direction (Fig. 6; Table 1). By contrast, the location of all sources for MRCFs and s1/4 in SEFs nearly overlapped in the same precentral region (Fig. 6; Table 1), whereas there was an apparent disagreement of source orientations between them (Table 2). This may refect the differentiation of neuronal assemblies in response to different kinds of afferent inputs, for example, the sm1 for MEFI was elicited by the natural finger movements, whereas s1/4 in SEFs was elicited by median nerve stimulation. Although both are the first cortical responses triggered in the periphery, different afferent inputs may contribute to the generation of these two types of source response.

### Relation of MRCFs to EMGs

To control rapid, self-terminated movements about a single joint, the activities of antagonist muscles toward the movement end are needed not only for braking ongoing movement (Marsden et al. 1983; Mustard and Lee 1987), but for end-point precision (Suzuki et al. 2001). However, neither of these was needed in our task. Instead, the complete relaxation of antagonist muscles was needed immediately after a pulsatile command had been issued. Therefore, even if the MEFI might be attributable to the reafferent signal from the periphery as suggested above, this MEFI component is not linked to the generation of reflexive muscle responses. MacKinnon et al. (1994) have examined an experimental situation in which a load compensatory reaction is or is not needed in the stretched wrist muscles. They found that the magnitude of EMG responses was modulated with task instruction, being largest with active and smallest with passive resistance. By contrast, the magnitude of the early evoked potentials, the dipole generator for which was confirmed to be in the deep layers of area 4, did not change across tasks. They suggested that instruction-dependent modulation of muscle responses occurs downstream from inputs to the primary motor cortex. The apparent disparity between postmovement MEFI response and muscle activities we found may be explained similarly.

The presence of MEFII and MEFIII components has been reported in several studies (Nagamine et al. 1994; Hoshiyama et al. 1997; Kristeva-Feige et al. 1997; Cheyne et al. 2006), but few studies have provided precise estimates for the source location of these components and their physiological significance remains largely unknown. Using an event-related beam-forming approach, Cheyne et al. (2006) have shown that the MEFII component reflects a second activation of the precentral gyrus in close vicinity to the anterior wall of the central sulcus, implying that this component reflects motor outputs relating to the control of ongoing movement such as contraction of the first antagonist muscles or subsequent second agonist activation. However, under the present task, activation of antagonist muscles was not required as discussed above and, in fact, compound spike potentials from the antagonist muscles were weak (Fig. 4). Therefore, the MEFII and perhaps also the MEFIII response, seem to be independent of the generation of control actions of antagonist muscles. The apparent disparity between MEFs and muscle excitations may reflect the independence of neuronal activities in the motor cortex from muscle excitations following the first agonist burst. Following the first agonist burst, the central generation of subsequent control actions for antagonist muscles may shift from cortical to subcortical system dependence (Flament and Hore 1986; Hore et al. 1991). Among many possibilities, the cerebellum may subserve the optimization of ongoing movements following first agonist activity by using sensory information (Jueptner et al. 1997; Schwarz and Their 1995; see also MacKinnon and Rothwell 2000).

### The neural basis of the MRCF waveform

In our movement task, reciprocal drive was not given to antagonist muscles, whereas the MRCFs exhibited their own rhythm independently of antagonistic muscles' activation, suggesting that a series of activations arises in an area in the precentral gyrus without inputs from the periphery for the second or third MRCF components. Here, we would like to briefly discuss the mechanisms underlying this finding. The intrinsic properties of cortical neurons and/or the resonant neuronal circuits among many cortical and subcortical areas may underly the generation of an alternating pattern of MRCF waveforms.

Extracellular field potentials are generated by neuronal dipoles created within elongated dendritic fields, aligned in parallel arrays. Cortical pyramidal cells with their long apical dendrites are the typical example of dipole generators. The current sink is the site of net depolarization, and the source is the site of normal membrane polarity or of hyperpolarization. In this scheme, an alternating waveform in the MRCFs can be regarded as corresponding changes in sink–source configuration along apical dendrites, leading to sequential changes in direction of the ECD's intracellular current. The current directed to the brain surface or superficial layer is thought to reflect the depolarization of proximal apical dendrites, whereas the current in an opposite direction is thought to be a surface reflection of the depolarization of the distal apical dendrites (Landau 1967; Schlag 1973; Wood and Allison 1981). Under this condition, successive changes in sink–source configuration may occur. Actually, in animal studies, the presence of this sequential reversal of sink–source configuration is commonly suggested in the somatosensory, visual, and auditory cortices (Towe 1966; Schlag 1973; Mitzdorf 1985). In human MEG studies, very similar polarity-reversed sequential activations in a cortical area have been shown among the somatosensory (Inui et al. 2004), nociceptive (Inui et al. 2003), auditory (Inui et al. 2006), and visual (Inui and Kakigi 2006) systems, suggesting the existence of a common intralaminar processing for feedforward sensory pathways. Therefore, such a common laminar mechanism is possibly present in the motor cortex and contributes to the successive reversals of ECD direction in this study.

The source activity used to model MRCFs in this study was apparently alternating in the anterior/posterior direction in cortical space (Fig. 1B). Based on our single-dipole assumption for composing MRCFs, it is suggested that the intracellular current for the first premovement component MF was directed anteriorly. This is consistent with the previous observation that excitation of motor cortex neurons preceding movement originates in the superficial cortical layer of the anterior wall of the central sulcus (Roland 2002; Larkum et al. 2004). Thereafter, our results suggest that the intracellular current for the first postmovement component MEFI is directed posteriorly (Fig. 1B-c). Given that the MEFI component is driven by muscle spindle signals which depolarize the proximal apical dendrite of motor cortex neurons via the thalamocortical projections (Rosen and Asanuma 1972; Lemon et al. 1976; Evarts and Fromm 1977; Wong et al. 1978; Lemon 1981), a posterior direction current may happen as shown in Figure 1B-c (see also Fig. 3).

Another possibility for the alternating waveform in MEFs may be found in the fact that the pyramidal neurons of motor areas are under the control of two different types of thalamocortical afferents. Motor thalamic nuclei, mainly composed of ventral anterior (VA) and ventral lateral (VL) nuclei, receive massive afferents from the basal ganglia and cerebellum and project their axons to motor cortical areas (for a review, see Groenewegen and Witter 2004; Jones 2007). These two forms of information are differentially supplied to distal and proximal apical dendrites, respectively, of cortical pyramidal neurons. Studies in animals have provided evidence of at least two types of VA–VL neurons; one *fast*-conducting type of cerebellar afferent-receiving neurons in the VA–VL complex sent their axons only to deep cortical layers, whereas the other *slow*-conducting type of cerebellar and/or pallidal afferent-receiving neurons sent axons to layer I (Sasaki et al. 1976; Sasaki and Gemba 1981, 1982; Jinnai et al. 1987). These two layer-specific differentiations of thalamocortical inputs may contribute to generate an alternative waveform of MEFs, such that once the motor cortex neurons are driven by some strong afferent volley originated in the periphery as expected in MEFI, the thalamocortical network entrains grouped behavior of these two regions to resonate for a short while.
